# Heart failure with preserved ejection fraction as a systemic inflammatory–metabolic disorder: through the cardio–hepato–pancreatic axis lens

**DOI:** 10.3389/fcvm.2026.1806841

**Published:** 2026-04-28

**Authors:** Han Naung Tun

**Affiliations:** 1 Office of MD Research, Geisel School of Medicine, Dartmouth College, Hanover, NH, United States; 2 Department of Medical Science, Bouvé College of Health Sciences, Northeastern University, Boston, MA, United States

**Keywords:** cardio-hepato-pancreatic axis, cardio-metabolic dysfunctions, heart failure with preserved ejection fraction (HFpEF), multi organ crosstalk, systemic inflammation

## Introduction

Heart failure with preserved ejection fraction (HFpEF) accounts for approximately half of all heart failure cases and is increasing in prevalence worldwide (1). Despite this burden, therapeutic progress has lagged behind that seen in heart failure with reduced ejection fraction. One explanation for this disparity may be conceptual rather than pharmacologic. HFpEF has historically been defined as a myocardial disorder characterized by impaired relaxation, increased ventricular stiffness, and elevated filling pressures. However, this paradigm has failed to yield consistent therapeutic benefit, raising questions about whether the heart is the appropriate primary target in HFpEF. Accumulating experimental, clinical, and epidemiological data increasingly support the notion that HFpEF is a systemic disease, arising from chronic inflammatory and metabolic stressors that converge on the cardiovascular system (1, 2). Having said that, the heart may represent an end-organ rather than the origin of disease. The newly proposed pathway that HFpEF is best understood through a cardio–hepato–pancreatic inflammator*y* axis, in which dysfunction of the liver and pancreas contributes directly to cardiac pathology (3).

## Limitations of the cardiac-centered model

The conventional framework of HFpEF emphasizes ventricular mechanics, neurohormonal activation, and pressure–volume relationships. While these features are undeniably present, they do not adequately explain several defining characteristics of HFpEF, including its strong association with obesity, diabetes, metabolic liver disease, frailty, and systemic inflammation. Moreover, therapies targeting myocardial contractility, fibrosis, or neurohormonal signaling have largely failed to improve outcomes, suggesting that myocardial dysfunction may be downstream of broader systemic processes (1, 2). Hepatic congestion is frequently observed in HFpEF, particularly in the setting of pulmonary hypertension and right ventricular dysfunction (2). Traditionally viewed as a passive consequence of right-sided heart failure, chronic hepatic congestion may instead play an active role in disease progression. Sustained elevation of hepatic venous pressure leads to sinusoidal dilation, hepatocellular hypoxia, and progressive fibrosis, a condition referred to as congestive hepatopathy (1–3). While early stages may be reversible with effective unloading, persistent congestion can progress to cardiac cirrhosis (4). Importantly, the congested liver becomes a source of inflammatory mediators, altered hepatokine secretion, and endothelial dysfunction, which may further promote myocardial fibrosis and microvascular injury. Within this framework, cirrhotic cardiomyopathy may represent a clinical manifestation of HFpEF rather than a distinct disease entity (4, 5).

## Systemic inflammation: insights from heart-pancreas interaction

The heart–pancreas interaction is particularly evident in acute pancreatitis, which induces a robust systemic inflammatory response characterized by elevated levels of IL-1β, IL-6, and TNF-α (6). These mediators can impair myocardial relaxation, disrupt endothelial function, promote microvascular ischemia, and precipitate arrhythmias. Patients with HFpEF, already characterized by limited diastolic reserve and coronary microvascular inflammation, may be especially susceptible to these inflammatory insults (6–8). Observational studies consistently demonstrate increased mortality and worse outcomes among patients with pre-existing heart failure hospitalized with acute pancreatitis, underscoring the clinical relevance of this interaction (9–11). Pancreatic involvement in chronic heart failure has received comparatively little attention. Pancreatic exocrine insufficiency (PEI) has been reported in a substantial proportion of patients with chronic heart failure and may result from splanchnic hypoperfusion, venous congestion, autonomic dysregulation, and chronic inflammation (7). In HFpEF, PEI may contribute to malabsorption, micronutrient deficiencies, sarcopenia, and cachexia, which features increasingly recognized as central determinants of exercise intolerance and poor outcomes (7). These extracardiac manifestations are unlikely to be explained solely by myocardial dysfunction and highlight the importance of considering pancreatic involvement in HFpEF pathophysiology.

## MASLD, obesity, and the adipokine hypothesis

Metabolic dysfunction–associated steatotic liver disease (MASLD) further strengthens the cardio–hepato–pancreatic model. MASLD serves as a chronic source of systemic inflammation, oxidative stress, and insulin resistance, independently increasing cardiovascular risk (12, 13). The adipokine hypothesis by Milton Packer also provides a mechanistic framework linking obesity to HFpEF, as dysfunctional visceral and epicardial adipose tissue secretes pro-inflammatory adipokines, such as leptin and resistin, while suppressing protective adiponectin (14). These changes promote myocardial fibrosis, ventricular stiffening, and endothelial dysfunction (12–14). From this perspective, cardiometabolic HFpEF is best conceptualized as an inflammatory–metabolic endotype rather than a purely clinical phenotype (14).

## Implications for clinical practice and research

Recognizing HFpEF as a systemic disorder challenges the adequacy of heart-centric management strategies. Interventions targeting congestion, inflammation, and metabolic dysregulation across organs may yield greater benefit than therapies focused solely on ventricular mechanics (15). The clinical efficacy of sodium–glucose cotransporter-2 inhibitors (SGLT2i) in HFpEF supports this paradigm, given their pleiotropic effects on inflammation, volume status, hepatic metabolism, and pancreatic stress. Similarly, glucagon-like peptide-1 receptor agonists (GLP1-RA) may favorably influence the cardio–hepato–pancreatic axis (16). Identification and treatment of PEI represent additional low-risk strategies deserving prospective evaluation.

Since HFpEF may be conceptualized as an inflammatory–microvascular disorder, in which chronic systemic inflammation drives coronary microvascular dysfunction, endothelial nitric oxide deficiency, and increased myocardial stiffness through collagen deposition and titin alteration, with key pathways involving IL-1β, IL-6, and TNF-α that represent promising targets for future therapies (17). While HFpEF has traditionally been viewed as a primary myocardial disorder, an increasing body of experimental and clinical evidence suggests that it frequently develops in the setting of systemic cardiometabolic and inflammatory stress (18–20). Within this framework, the myocardium may represent an important target organ of broader systemic pathology rather than the sole origin of disease (18–20).

Patients with HFpEF should receive care across cardiology, hepatology, and gastroenterology services without an integrated conceptual framework (21). A systems-based, multidisciplinary approach incorporating biomarkers, advanced imaging, and nutritional assessment may allow earlier identification of patients engaged in this inflammatory tri-organ loop and enable more personalized interventions. The concept of multi-organ interaction in cardiovascular disease is well established, as illustrated by the reno-cardiac syndrome (21, 22). Similarly, interactions between the heart and other visceral organs including the liver and pancreas, which in turn may contribute to the pathophysiology of HFpEF through shared inflammatory, metabolic, and neurohormonal pathways. The proposed cardio–hepato–pancreatic axis should therefore be viewed as one potential framework within a broader network of systemic organ crosstalk influencing HFpEF development (23). Future research should focus on mechanistic studies and biologically informed phenotyping to identify patients most likely to benefit from targeted therapies addressing this axis.

## Conclusion

HFpEF should be reconsidered as a systemic inflammatory–metabolic disorder with cardiac manifestations, rather than a disease confined to the myocardium. The cardio–hepato–pancreatic axis provides a coherent and biologically plausible framework that reconciles the heterogeneity, comorbid burden, and therapeutic challenges of HFpEF. Embracing this perspective may be essential for advancing beyond the therapeutic stagnation that has long characterized this condition. Accordingly, it is strongly suggested that heart failure investigators include pancreatic evaluation and portal hemodynamic validation in future HFpEF research protocols.
